# Probiotication of Nutritious Fruit and Vegetable Juices: An Alternative to Dairy-Based Probiotic Functional Products

**DOI:** 10.3390/nu14173457

**Published:** 2022-08-23

**Authors:** Floyd Darren Mojikon, Melisa Elsie Kasimin, Arnold Marshall Molujin, Jualang Azlan Gansau, Roslina Jawan

**Affiliations:** Biotechnology Programme, Faculty of Science and Natural Resources, Universiti Malaysia Sabah, Jalan UMS, Kota Kinabalu 88400, Sabah, Malaysia

**Keywords:** lactic acid bacteria, probiotication, plant-based juices, functional and nutraceutical food

## Abstract

Fruits and vegetables are widely known to be rich in nutrients, antioxidants, vitamins, dietary fiber, minerals, and a bioactive molecule, making them an essential component of a balanced diet with multiple documented positive effects on human health. The probiotication of plant-based juices for the production of functional and nutraceutical food serves as a healthy alternative to dairy probiotics. They are cholesterol free, lack several dairy allergens, and also encourage ingestion for people with lactose intolerance. This review highlights valuable claims regarding the efficacy of different probiotic strains on various diseases. A comprehensive nutrition comparison and the preference of plant-based over dairy probiotic drinks is also discussed, supported with updated market trends of probiotic drinks (dairy and non-dairy based). An extensive compilation of current plant-based probiotic drinks that are available in markets around the world is listed as a reference. The fermentability of carbon sources by probiotic microorganisms is crucial in addressing the development of plant-based drinks. Therefore, the pathway involved in metabolism of sucrose, glucose, fructose, and galactose in fruit and vegetable juice was also underlined. Finally, the key factors in monitoring the quality of probiotic products such as total soluble solids, sugar consumption, titratable acidity, pH, and stability at low storage temperatures were outlined.

## 1. Introduction

Probiotics are beneficial microorganisms that can exert therapeutic effects on the organisms ingesting them [1]. It is recommended to consume a minimum of 10^6^–10^7^ CFU/mL daily to experience the health benefits of probiotics [2,3]. Among the beneficial health claims of probiotics are the prevention of diarrhea, alleviation of gastric discomfort, relief of inflammation, and improvement of immunity [4,5,6,7]. The probiotication of beverages can increase their market value due to the presence of beneficial bacteria [8]. Fermentation using probiotic strains could improve the aroma and taste profile and increase the shelf-life, as the cell culture breaks down fermentable sugars to release by-products such as lactic acid, which has antimicrobial properties, and modifies the final product to have a tangy and sour taste [9].

**Figure 1:**
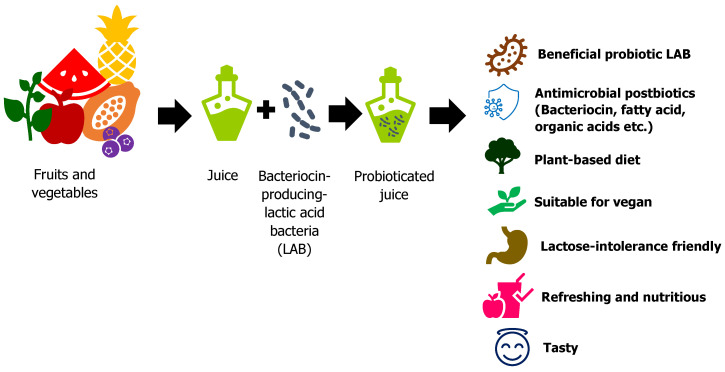
Stages in probiotication of fruits and vegetables juice.

Up until now, the global probiotic drink market has been dominated by dairy-based beverages [10]. However, in recent years, the demand for plant-based products is growing at a faster rate, with an increased demand for the incorporation of probiotics in fruit and vegetable juices. The increased awareness of the allergenicity and high levels of cholesterol and fats in milk has incited a shift in the preference of consumers to healthier alternatives [11], as even soybean products could also impose on the soy allergies of some consumers [12]. Furthermore, lactose-intolerance and vegetarian, animal, and environmental activism have driven companies to expand their businesses into dairy-free and vegan-based drinks. Thus, the utilization of fruits and vegetables to make probiotic drinks must be developed to meet the demand for dairy alternatives and vegan-based drinks. The summary of stages involved in the production of non-dairy-based probiotic products and the benefits of probioticated plant-based juice is shown in Figure 1.

Lactic acid bacteria (LAB) are bacteria that produce lactic acid as the by-product of the lactic acid fermentation of a substrate [13,14]. Bacteria from the genus *Lactobacillus* and *Bifidobacterium*, both widely applied as probiotics, are the main inhabitants of the intestine [15] and are able to produce antimicrobial substances as well as contain fewer pathogenic strains [16]. Despite being used to ferment milk products, which mainly contain lactose, lactobacilli can also utilize various types of carbon sources such as glucose, galactose, sucrose, and maltose [17]. Practically, it is possible to maintain the minimum daily probiotic dose during the fermentation and refrigeration of LAB strains in non-dairy juices. Yuliana et al. [18] reported that the fermentation of coconut milk by *L. acidophilus* resulted in 10^9^ CFU/mL viable cells after 28 h, and the samples, which were stored at 5 °C, subsequently retained a viable cell count around 10 log CFU/mL on the 16th day, with a significant decrease in pH and sugar content and an increase in acidity and total soluble solids. Meanwhile, Yoon et al. [19] revealed that *L. casei*, *L. plantarum*, and *L. delbreuckii* increased in viable cell count after 72 h of fermentation (around 10^8^ CFU/mL); however, during the refrigeration period, only *L. plantarum* retained the highest viability (10^7^–10^8^ CFU/mL), while *L. delbreuckii* reduced in viability to 10^5^ CFU/mL at week 3 and week 4, and *L. casei* lost its viability after 2 weeks of refrigeration. It is believed that the wide range of fermentable sugars such as glucose, fructose, galactose, sucrose, mannitol, stachyose, and raffinose in fruits and green plants provide multiple options for probiotics to utilize and grow [20,21].

## 2. Probiotics

‘Probiotics’ is defined by the Food and Agriculture Organization of the United Nations (FAO) and World Health Organization (WHO) as “live microorganisms which when administered in adequate amounts confer a health benefit on the host” [1]. Two of the most widely used probiotics are from the genera *Lactobacillus* and *Bifidobacterium*, as both constitute most of the normal intestinal microbiota in various mammalian species [15]. Right after birth, maternal microbiota will colonize the gastrointestinal tract (GIT) in which the first colonizers are facultative anaerobes such as *Lactobaciili*, *Enterococci*, and *Enterobacterium*. Anaerobic bacteria such as *Bifidobacterium*, *Bacteroides*, and *Clostridium* will colonize the GIT with an increase in age and compete with the facultative anaerobes, setting the colon environment to favor anaerobe growth over time [22].

More than 400 bacterial species can be found in a normal adult human GIT. Surprisingly, 50% of the wet weight of human feces is contributed to by bacterial biomass. The intestinal microflora maintain their population density by preventing opportunistic colonization by pathogenic bacteria with a mechanism referred to as “colonization resistance” or “barrier effect” [23,24]. Therefore, probiotics are beneficial bacterial supplements, usually in a dried form or as live culture, and, when ingested as food, can assist the preventive mechanism of the host’s gut microbiota against bad microorganisms.

### 2.1. Beneficial Claims of Probiotics

Research demonstrating the beneficial effects of probiotics to human health has been widely published. Probiotics must grow and/or perform therapeutic activity to benefit the host [25,26]. Although the mutualistic effect of probiotics is yet to be elucidated, their possible inhibitive mechanism may include the production of antimicrobial compounds, the competitive exclusion of pathogen binding, competition for nutrients and space, and the modulation of the host’s immune system. As shown in Table 1, the beneficial claims of probiotics include the reduction in infectious diarrhea and allergic reaction, alleviation of gastritis, alternative cancer remedy, modulation of immune cells, and bacterial vaginosis treatment.

Park et al. [7] reported that children with infectious diarrhea caused by rotavirus have shown a reduced frequency of diarrhea and vomiting after three days of oral administration of s powdered probiotic formula containing 20 × 10^9^ CFU/g of *Bifidobacterium longum* BORI and 2 × 10^9^ CFU/g of *Lactobacillus acidophilus* AD031 compared to the placebo treatment (probiotic-free skim milk). However, the underlying therapeutic mechanism is yet to be investigated. According to an in vitro study conducted by Wang et al. [5], it was revealed that yoghurt supplemented with *Bifidobacterium lactis* Bb12 at a minimal viable cell count of 9 × 10^8^ CFU/mL suppresses the growth of *Helicobacter pylori* and decreased the C-urea breath test (UBT) value, suggesting the decreased urease activity of *H. pylori*. This finding is supported by Midolo et al. [27], whereby lactic acid produced by *Lactobacillus* sp. strongly inhibited the growth of *H. pylori* NCTC 11637 in vitro in a pH- and concentration-dependent manner. Coconnier et al. [28] found a decrease in urease activity and cell viability of *H. pylori* with no gastric histopathological lesions in human muco-secreting HT29–MTX cells after the administration of the *Lactobacillus acidophilus* strain LB spent culture supernatant (LB–SCS).

Ingestion of a minimum dosage of probiotics is recommended to bestow health benefits on the host. As such, manufacturers should indicate a minimum daily dosage and expiry date for each strain of their probiotic products, supported by strong scientific evidence and sales approval [1]. The dosage, however, varies in opinion among scientists. According to Sanders [29], a range of doses between 10^9^ and 10^11^ CFU/mL of probiotics is the effective daily dose. Furthermore, Martins et al. [2] and Shori [3] agreed that 10^6^–10^7^ CFU/mL should be the minimum number of viable probiotic cells to be ingested daily. Recently, Gangwar et al. [30] suggested that a probiotic product with 10^6^–10^8^ CFU/mL or g of cells can exert therapeutic effects and meet the daily requirement of probiotics.

Moreover, a person with a gastrointestinal disease such as acute diarrhea should ingest a larger dose of probiotics daily. Basu et al. [31] stated that ingesting 10^10^ to 10^12^ CFU of *Lactobacillus rhamnosus* GG daily was effective in treating acute diarrhea compared to 10^7^ CFU/day. A minimum dose of 10^8^–10^9^ CFU/day showed a reduce rotavirus concentration in patient fecal matter [32,33]. In contrast with the positive findings, a supplementation of 10^8^ CFU/day of *S. thermophilus* and 10^9^ CFU/day of *B. lactis*, however, did not reduce the duration of rotavirus diarrhea [34]. This suggests that there is no definite minimum daily dose of probiotics required for daily ingestion, as varying factors such as probiotic strain, type of causative pathogen, severity of symptoms, age, and race can render the therapeutic effects of probiotics ineffective.

Probiotic administration can regulate the neuropsychological functions of the central nervous system. The bidirectional gut–brain relationship can be influenced by the population of gut microflora. A study by Liu et al. [35] revealed that mice supplemented with probiotics have reduced depression compared to non-probioticated mice. Under stress conditions, one group of mice is supplemented with fluoxetine hydrochloride and another group of mice is given multi-strains of probiotic daily for 8 weeks. The mice subjected to the probiotics showed less depressive-like behavior due to the lowered corticosterone levels in their blood serum. The probiotic-treated mice showed a higher number of fecal microbiotas, suggesting that the probiotics may have released certain compounds capable of lowering the mice’s blood corticosterone levels [35].

Administration of *Lactobacillus* sp. decreases self-injurious behavior (SIB) in primates. SIB is a complex phenotype that occurs in 10–12% of non-human primates and 7–34% of humans. The condition is caused by abnormally elevated hypothalamic-pituitary-adrenocortical (HPA) axis activity, which causes primates to self-harm such as biting fingers or other body parts during sleeping. The injuries sustained may cause infection in the wild. According to research by McGinn [36], the rhesus macaque monkeys with SIB supplemented with *Lactobacillus reuteri* containing an average 200 million CFU/tablet modestly decreased biting behavior. Therefore, the sleeping quality of the monkeys in the SIB group is improved.

### 2.2. Probiotic Attributes

To exert their beneficial effects on the host, probiotic strains need to pass through the harsh conditions of the GIT. Gastric-acid and bile-acid resistance are two main criteria measured in selecting viable probiotic strains. Food travels along the digestive tract starting from the mouth, esophagus, stomach, small intestines, and lastly the large intestine [37]. The stomach is considered hostile to most bacteria. The parietal cells release gastric juice in response to a histamine released by enterochromaffin-like cells in the presence of gastrin [38]. Gastric juice contains hydrochloric acid (HCl) which causes the pH of normal adult stomach fluid to be acidic (1.5 to 3.5) (Halperin, n.d.). The acidic condition is optimal for pepsin activity. Within 15 min, most bacteria die in the presence of HCl and pepsin at pH levels lower than 3.0 [39].

Depending on the species and strain, acid sensitivity was observed in most microorganisms at pH levels below 3.0 [40,41]. Tennant et al. [42] revealed that the Gram-negative pathogenic bacteria, *Y. enterocolitica* 8081u^−b^ (mutant), dramatically reduced in viability from 114.9 at pH 7.0 to 12.3 at pH 3.5, respectively, with all cells wiped out below pH 3.0. In contrast, the wild-type *Y. enterocolitica* 8081^b^ also experienced reduced cell viability but was still present even at pH 2.0. The acid resistance of the wild type contributed to its ability to produce urease, while the urease mutant cannot synthesize urease, causing it to be susceptible to acid [42]. While stomach acid is an effective evolutionary mechanism to inhibit the growth of pathogens, beneficial microbes can be killed as well.

Food typically stays in the stomach for around 2 to 4 h before being emptied out during gastric digestion [43]. *Lactobacillus* species, one of the main inhabitants of the colonic compartment, are essentially resilient to stomach acid. The human-derived strain *L. rhamnosus* GG is a commercial probiotic strain that has been shown to survive passage through the highly acidic stomach with a tolerance to acid as low as 2.5 for 4 h [44]. It was thought that Gram-positive bacteria express a multiple-subunit membrane-bound ATP synthases called F_0_F_1_-ATPase as a shielding mechanism against acidic conditions [45]. The F_0_F_1_-ATPase consists of two protein portions of F_1_ and F_0_. The F_1_ portion consists of α, β, γ, δ, and ε subunits responsible for the catalyzation of ATP hydrolysis. The F_0_ is the integral membrane portion that contains a, b, and c subunits which form membranous channels for proton translocation [46]. The F_0_F_1_-ATPase is expressed at low pH levels and generates a proton motive force via portion expulsion, which reduces cytoplasmic H^+^ concentration, leading to an increased intracellular pH at low extracellular pH [47]. Cocoran et al. [48] proved that fermentable sugars in food can assist the survival of *L. rhamnosus* GG in an acidic environment as the probiotic metabolize the sugar through glycolysis to provide sufficient ATP reserves for F_0_F_1_-ATPase function in pH homeostasis.

Bile acid is produced by hepatocytes through daily cholesterol degradation at approximately 350 mg [49] to remove harmful metabolic waste substances such as bile salts, bilirubin phospholipid, cholesterol, heavy metals, and toxins [50]. Bile salts form a major part of this complex aqueous secretion and function to emulsify fats from food for easier absorption. In addition, bile salts also act as antimicrobial agents to control the population of intestinal microbiota. Bile inhibits the growth of bacteria by disrupting their cellular membranes [51], inducing DNA damage [52], misfolding proteins [53], and chelating iron and calcium [54,55].

**Table 1:** Beneficial claims of probiotics.

Beneficial Claims	Probiotic Treatment	Main Findings	Reference
Prevention of infectious diarrhea	*Bifidobacterium longum* BORI and *Lactobacillus acidophilus* AD301	Reduced duration of rotavirus diarrhea in young Korean children.	Park et al. [7]
Alleviate symptoms of type B gastritis and peptic ulcers and prevention of gastric cancer	*Bifidobacterium lactis* Bb12	Growth inhibition of *Helicobacter pylori* leading to a decrease in urease activity, a key enzyme essential for survival of the pathogen in the stomach acid after 6 weeks of therapy.	Wang et al. [5]
Relieve inflammatory bowel disease syndromes	*L. casei*, *L. plantarum*, *L. acidophilus*, *L. delbrueckii* subsp. *bulgaricus*, *B. longum*, *B. breve*, *B. infantis*, and *Streptococcus salivarius* subsp. *thermophilus*	Restoration of microbial flora to normal level through observation of increased lactobacilli, bifidobacterial, and *Streptococcus salivarius* in patient’s fecal matter, leading to a reduced inflammation and symptoms of chronic pouchitis.	Gionchetti et al. [4]
	*Lactobacillus* GG	Significant reduction in Crohn’s disease activity and increased intestinal permeability after 4 weeks medication of *Lactobacillus* GG enterocoated tablets containing 10^10^ CFU/g.	Gupta et al. [56]
Alternative prevention for cancer	*Lactobacillus rhamnosus* strain GG and LC-705	Decrease in carcinogenic aflatoxin level in the chicken lumen after daily ingestion probiotic strains.	El-Nezami et al. [57]
Modulate host’s immunity	*L. acidophilus*, *L. casei*, *L. reuteri*, *Bifidobacterium bifidum*, and *Streptococcus thermophilus*	Induced hyporesponsiveness of T- and B-cells, non-apoptotic downregulation of T helper (Th)1, Th2, and Th17 cytokines, and generation and increased suppressor activity of CD4^+^CD25^+^Tregs.	Kwon et al. [6]
Allergy prevention and treatment	*B. longum* NCC 3001 and *Lactobacillus paracasei* NCC 2461	Downregulation of allergen-specific immune responses contributing to airway inflammation in mucosal lining of polysensitize mouse.	Schabussova et al. [58]
	*Bifidobacterium lactis* Bb-12 and *Lactobacillus* strain GG	Improved skin condition in infants suffering atopic eczema after 2 months supplementation of the probiotic formulas.	Isolauri et al. [59]
Bacterial vaginosis treatment	Yoghurt (containing mostly *Lactobacillus* sp.)	Cured bacterial vaginosis after 1 to 2 months of intra-vaginal treatment through the increased lactobacilli flora and vaginal pH correction.	Neri et al. [60]

To counteract the detergent activity of bile salts, enteric bacteria develop bile resistance through the enhancement of the cell membrane by: (1) expression of long O-antigen chains in the lipopolysaccharides [61]; (2) expression of AcrAB-TolC efflux system where the TolC protein channel, AcrB transporter, and AcrA periplasmic protein act together to pump out bile salts from the cytoplasm [62,63]; and (3) performance of DNA repair mechanisms such as SOS—associated DNA repair, recombinational repair by the RecBCD enzyme, and base excision repair (BER) in response to bile-induced DNA damage [64]. Nevertheless, tolerance to bile salts is also species- and strain-dependent, as reported by Davati et al. [65] who discovered that *P. pentosaceus* has more than a 90% survival rate compared to *L. mesenteroides* (less than 40%) after 6 to 24 h of incubation with 0.4% (*w*/*v*) bile salts.

### 2.3. The Market of Probiotic Drinks

Probiotication is a process of inoculating beneficial microorganisms (mostly lactic acid bacteria) into a liquid substrate to manufacture functional beverages, which subsequently adds market value due to the various health benefits of probiotics [66]. Changes in pH, sugar content, acidity, and viable cell count are observable on various types of raw materials when applying probiotics as the fermenter [30,67,68,69]. Basically, probiotic drinks can be made up of dairy- and non-dairy-based ingredients. In 2019, the global market size of probiotic drinks was worth USD 13.65 billion, and its compound annual growth rate (CAGR) is expected to increase by 6.1% from 2020 to 2027. Nonetheless, the market of probiotic drinks is still dominated by dairy-based drinks, which held more than 55% of the revenue share in 2019 [10].

On the other hand, the fastest market growth was seen in the plant-based product segment over the forecasted period. There is a significant increase in the demand for plant-derived drinks, including probiotic fruit and vegetable juices [70]. The driving force of the shift towards a non-dairy-based product are lactose-intolerant, vegetarians, and animal-lover consumers [10]. On top of that, the increasing awareness of the detrimental effects of milk products such as high cholesterol and fat content and the presence of allergens may discourage a lot of milk-lovers [11].

## 3. Dairy and Non-Dairy Based Probiotic Drinks

A dairy-based probiotic drink is a milk supplemented with probiotics [71]. The milk is mostly sourced from cows, but other animal sources such as goats, sheep, and water buffalo milks can also be used [72]. A typical production procedure involves pasteurization, where the milk is heated to 71.7 °C for 15 to 25 s, followed by a brief and immediate cooling below 3 °C to extend the shelf-life by inactivating the spoilage microorganism and its enzymes to preserve the nutritional value, before being aseptically inoculated with the probiotic strain for fermentation. A non-dairy based probiotic drink uses non-milk substrates such as fruits, vegetables, and oatmeal [73]. Similar to milk processing, the non-dairy substrate needs to be sterilized by autoclaving at 121 °C at 15 psi for 15 min [30] prior to the fermentation process.

Milk is a nutrient-dense substrate which provides a sufficient supply of carbon (lactose), nitrogen (casein and whey), and mineral sources (calcium, phosphorus, sodium, and potassium) [72,74] for the probiotics to grow. In contrast, non-dairy substrates made up of fruits and vegetables possess a wide range of nutritional composition, depending on the maturation stage of the fruit and the parts, species, and variety of the fruit and vegetables themselves. Lim et al. [75] compared the nutrient composition of cempedak fruit, *Artocarpus champeden,* and its hybrid *Nanchem* and found that the flesh of *A. champeden* contained higher total carbohydrates (16.2–28.3 g/100 g) compared to *Nanchem* (7.5–30.0 g/100 g). The crude protein, fat, and ash contents of *A. champeden* flesh were also relatively higher than *Nanchem*. In terms of the maturation stage, the unripe *A. champeden* fruit contained a higher percentage of ash (4.6–5.0%), crude fiber (12.9–23.9%), crude protein (7.3–15.9%), and crude fat (3.9–6.4%) than the ripe flesh. However, the ripe *A. champeden* fruit stored higher total carbohydrates (16.2–28.3 g/100 g) than the unripe flesh (2.4–5.1 g/100 g).

### 3.1. Cholesterol and Fat Content

Eating a diet with high fat and cholesterol greatly increases the risk of developing cardiovascular diseases [76]. The World Health Organization stated that the main cause of global death is cardiovascular diseases. Annually, around 17.9 million people die due to heart-related complications [77]. The complex dairy fat is made up of 400 types of fatty acid species, where 65–70% of it is saturated fatty acids (SFA) [78]. Faye et al. [79] discovered that fresh cow’s milk contains relatively higher cholesterol levels (8.51 mg/100 g) compared to camel’s milk (5.64 mg/100 g). On top of that, the fat content of cow’s milk is also higher (4.52 g/100 g) than camel’s milk (2.69 g/100 g). Cholesterols are only synthesized in animals, including humans [80]. Therefore, cholesterol is only sourced from animal and dairy-based products, whereas plant-based foods such as fruit, vegetables, nuts, and grains are free of cholesterol (Heart UK, n. d.). The University of California San Francisco (UCSF) Health [81] recommends an intake of not more than 300 mg of cholesterol per day.

Cholesterol is important for retaining the fluid mosaic model of mammalian cellular membrane, as it assists the lipid bilayer in terms of permeability, hydrophobicity, and fluidity [82]. However, an excessive intake of saturated fats and cholesterol causes an increase in low-density lipoprotein (LDL) in the blood plasma, and the accumulation of these bad cholesterol leads to atherosclerosis, a condition where the artery experiences a blockage due to plaque formation, and the supply of oxygen to the heart is cut off [83]. This will result in the occurrence of heart attacks and, even worse, can lead to death. In developing countries, atherosclerosis is reported as the major cause of mortality [84]. Published data by WHO in 2017 revealed that 22.13% of total deaths in Malaysia were caused by heart attacks, and the rate increased to 24.69% in 2018 [85].

Excessive fatty food consumption along with a sedentary lifestyle and genetics contribute to overweightness and obesity [86]. The Cleveland Clinic recommends an adult only consume fats around 20% to 35% of their daily total calories. Furthermore, to reduce detrimental health issues, it is suggested to only consume 15% to 20% monosaturated fats, 5% to 10% polysaturated fats, <10% saturated fats, and zero trans-fat out of the recommended daily intake in fat percentage [87]. Overweightness and obesity are associated with various medical complications such as fatigue, diabetes, high blood pressure, heart disease, and several types of cancer [88]. In all plant-based products, the fats are predominantly unsaturated fats with a low percentage of saturated fats [89], making it a healthier alternative for functional beverages.

### 3.2. Allergens

Generally, two major milk proteins responsible for sparking an allergic response in humans are casein and whey. The acidification of the cow milk results in two fractions: Fraction 1 appears as solid coagulum (coagulated proteins) called casein contributes to 80% of the total milk protein, and Fraction 2 appears with a liquid consistency (lactoserum) called whey contributes to 20% of the total milk protein [90,91,92,93]. Allergens in the casein fraction comprised 32% αS1-casein, 28% β-casein, 10% αS2-casein, and 10% K-casein [94]. Meanwhile, the whey fraction contains allergens such as α-lactalbumin (5%) and β-lactoglobulin (10%) [92,95], as well as traces of immunoglobulins, bovine serum albumin, and lactoferrin [96].

The allergens in milk exhibit immunoglobulin E (IgE) epitope clusters [97]. The IgE epitopes cluster in the allergen aids in the cross-linking between IgE antibodies and effector cells such as mast cells [98]. Then, the mast cells secrete histamine, an inflammatory mediator commonly associated with allergic responses, and promote vasodilation (dilating of blood vessel to deliver immune cells to the affected site) and tissue damage [99]. Gupta et al. [100] reported the 2.4% prevalence of a cow’s milk allergy (CMA) in consumers aged 18 to 29 years old in the U.S. Thus, non-dairy beverages are an excellent alternative for consumers with CMA. On another note, the use of soybeans to replace cow’s milk has also shown allergic reactions in consumers (allergic to soy) due to the soy protein [12]. Hence, fruits and vegetables are the best alternatives to dairy [73].

### 3.3. Consumers Preference on Non-Dairy Products

The preference of non-dairy- over dairy-based products is more prevalent among lactose-intolerant and vegetarian consumers. Animal abuse, environmental damage, and the search for new taste profiles drive consumers to resort to non-dairy products. Milk contains lactose (C_12_H_22_O_11_), a disaccharide made up of glucose and galactose subunits. Lactose makes up 2% to 8% of milk by weight [101]. To metabolize lactose, mammals, including humans, secrete a lactase enzyme called β-d-galactosidase which cleaves the glycosidic bonds between the glucose and galactose. However, the expression of lactase decreases with age as humans consume less milk throughout adulthood [102]. A meta-genomic analysis from Storhaug et al. [103] estimated that 68% of the world’s population is lactose-intolerant in which individuals from Africa and Asia form the major proportion. Meanwhile, approximately 36% of the United States (U.S.) population has lactose malabsorption problems, whereas ethnic and racial groups including African Americans, American Indians, Asian Americans, and Hispanics possess lactose-digesting problems [104].

Furthermore, awareness of animal cruelty in the dairy industry has also increased over the years due to the emerging animal rights movement and advancements in mass media [105]. The People for The Ethical Treatment of Animals (PETA) [106], stated that cows are kept in confined compartments and treated as machines that exclusively produce milk without meeting their most basic desires. Female cows are impregnated forcefully through artificial insemination right after delivering their infants [107], while calves are taken away from their mothers within 24 h of birth [108], and the milk intended to be consumed by the calves is harvested and sold as functional beverages [74]. On top of that, a cow that has been confined for 2 to 3 months generally develops mastitis, a severe dermal inflammation caused by the overgrowth of *E. coli* on the mammary glands [109].

Factory-farmed animals, including those in dairy farms, produce manure, which is extremely detrimental to the environment. The U.S. Department of Agriculture reported 1.5 billion metric tons of animal waste produced each year by the U. S. meat and dairy industries [110]. Eventually, these waste materials end up into waterways, polluting downstream rivers and lakes [111].

In addition to the above, commercialized fermented fruit and vegetable juices provide a wide range of taste profiles for all age groups of consumers compared to probiotic milk [73]. Not only are fruit and vegetable juices nutritious, but they are also highly refreshing and thus are suitable candidates in manufacturing a healthy functional drink [70].

### 3.4. Recent Development of Non-Dairy Probiotic Drinks

The global market for dairy alternatives is growing annually. In 2020, the global market for non-dairy-based products was USD 12,270 million, and this is forecasted to increase in CAGR from 2021 to 2026 by 11.0% [112]. The global food and beverage industry shows an increase in demand for dairy substitutes. Therefore, many companies have emerged to become the key players in the industry for non-dairy-based beverages. Lifeway, a company selling cultured milk kefir, expanded their market into vegan-based products through the invention of Plantiful, a probioticated pea juice [113]. Table 2 shows a list of commercialized non-dairy probiotic products corresponding to their manufacturer, probiotic strain, and the raw materials used.

Many manufacturers use a combination of multiplex probiotic strains, while some only use a single probiotic strain, e.g., NextFoods utilized only *Lactobacillus plantarum* LP299V to produce three distinct types of GoodBelly Juice Drinks [114]. The use of a single probiotic strain reduces interspecific competition for limited nutrients and space [115]. Nonetheless, multiple bacterial strains could coexist in the same media, as shown by Lifeway Foods—their Plantiful vegan-based drink utilized ten types of probiotics [116]. Griffin and Silliman [116] stated that multiple organisms can coexist through resource partitioning, where different forms of limited resources are slightly consumed, or the same limited resource is consumed at different times and locations to reduce interspecific competition. In addition to this, gut microflora coexist and perform competitive exclusion, a mutualistic mechanism to prevent the growth of invading microorganisms [117].

Melo-Bolivar et al. [118] discovered that bacterial gut microbiomes from a Nile tilapia inhibited the growth of the pathogenic *Streptococcus agalactiae*, and notable changes were observed in the dominant species in the culture (*Lactococcus* spp. replaced *Cetobacterium* and become the dominant species in the community). The ten probiotics in Plantiful belong to the genera *Lactobacillus*, *Bifidobacterium*, and *Streptococcus*, all of which comprised the normal intestinal microbiota in humans. Therefore, these probiotics can grow in parallel to one another in the same medium, similar to what complex indigenous microbiome performs in the human intestine [22].

Biomel, GT’s Organic Kombucha, KeVita Apple Cider Vinegar Tonics, and VitaCup Immunity Coffee Pods contain *Bacillus coagulans* [119,120,121,122], which can reproduce by spore formation. The use of spores is beneficial in the manufacturing and storage process of probiotic drinks, as the *B. coagulans* spores are resistant to heat, cold temperatures, and stomach acid and remain dormant in the juice but germinate once it passes through the gastric compartment [123]. While most companies utilized commercial probiotics, some used naturally growing bacteria from a fermented starter culture. GT’s Organic Kombucha, KeVita Apple Cider Vinegar Tonics, and Gut Shot^®^ applied starter cultures from kombucha (fermented tea), apple cider (fermented apple juice), and sauerkraut brine (fermented cabbage), respectively. The use of fermented substrates already enriched with microbial populations effectively shortens the fermentation period for the next batch of fresh substrate [124].

## 4. Comparison of Cereal and Fruit-Based Probiotic Beverages

Cereal-based probiotic drinks are basically the juice extracted from cereal products such as millet, oat, and rice that is fermented with a selected probiotic strain. Aside from fruit-and-vegetable-based probiotic beverages, researchers gear toward a cereal-based substrate to serve as a mode of transportation of probiotic strain into the human body. The edible endosperm of a cereal seed contains the ingredient to make a cereal juice. Compared to fruit, extracting cereal juice from the endosperm involves similar steps such as soaking in water for several hours (to increase water content and soften the seed), followed by draining (to remove excess water) and then wet milling (to separate the solid and liquid part). From here, the juice is introduced to heat treatment (e.g., pasteurization) before being inoculated and fermented with a pre-selected probiotic [125].

Cereal-based probiotic drinks differ from the ones made of fruit juices. One obvious difference is the production process. It is much easier to extract juice from cereal, as it comes from the endosperm of seeds. On the other hand, fruit juice requires extra processes if it comes from a coconut, which needs to be cracked open and grated. Some fruit source such as mango and papaya need only peeling and seed removal before cutting into smaller pieces. When comparing both cereal and fruit juices, the stability of both fermented drinks depends on the type of substrate and probiotic strain. Fruits generally contain more diverse groups of dietary fibers that serve as prebiotics for the good bacteria in the refrigeration period. However, upon the production process, the nutritional content of the cereal or fruit juice may be altered. The starch granules that present more highly in cereal endosperm may swell up upon heat treatment, causing water to be absorbed in an irreversible manner which leads to starch gelatinization. When the starch gelatinizes, the juice thickens and becomes less favorable to be used in beverages. Heating can also lead to the degradation of bioactive compounds in fruit juice, lowering their medicinal properties.

Fortunately, consumers can benefit more from drinking cereal- or fruit-based probiotic beverages compared to probioticated milk. Cereal- and fruit-based probiotic juices are healthier than an alternative to milk; therefore, they are less in fats and cholesterol with a handful amount of carbohydrates, proteins, dietary fibers, minerals, and vitamins. Moreover, consumers can have a wider selection of flavors, as the source of cereal and fruit-based beverages can vary, some having a unique taste and aroma. In addition, the viability of probiotic drinks in cereal and fruit-based beverages can suffice for the minimum probiotic dosage (above 6 log CFU/mL). Hassan et al. [125] reported that rice-based beverages can maintain a viable cell count around 8 log CFU/mL of *S.thermophilus*, *L. acidophilus*, and Bifidobacterium BB-12. Meanwhile, the *Lactobacillus casei shirota*, which is usually used in Yakult can also grow and survive in low temperature with a viable cell count around 10^9^ CFU/mL. Table 3 summarizes the advantages and disadvantages of cereal- and fruit-based probiotic beverages.

## 5. Utilization of Fermentable Sugars in Non-Dairy Substrates

Fermentable sugars are metabolizable sugars used by bacteria during the fermentation period. In plant-based matrices, bacteria can use sucrose, glucose, fructose, and galactose as simple sugars to thrive during the fermentation process, along with soluble fibers as prebiotics to sustain their cellular processes. A diverse range of fermentable carbohydrates in the diet can provide nutritional as well as potential health benefits to humans. The bacterial fermentation of fermentable sugars primarily results in the production of short-chain fatty acids (e.g., acetic, propionic, butyric acid, and lactic acid) that are beneficial for GIT health, such as promoting colonic health, as they are involved in the control of colonic mobility, colonic blood flow, and GIT pH, all of which affect nutrient and electrolyte absorption [21].

### 5.1. Production of Fermentable Sugar in Plants

**Figure 2:**
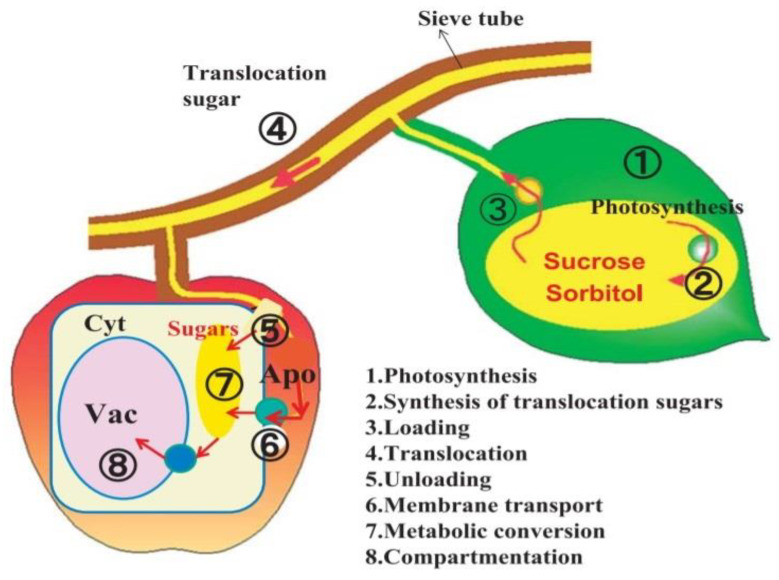
Movement of photoassimilate from the leaf to the fruit. Adapted from Yamaki, [132].

In plants, the translocation sugars is found in various types of horticulture crops, whereby sucrose is the main sugar translocated between the leaves and the fruits, and sugars such as sorbitol, raffinose, stachyose, and mannitol can be also found [20]. Sugar production in plants can be illustrated based on Figure 2. Plant leaves perform photosynthesis to produce photoassimilates, i.e., the biological compounds generated by assimilation in light-dependent reactions [126].

The energy-storing photoassimilate is converted into sucrose and sorbitol before being translocated in the phloem by a transporter. The translocation process applies the pressure-flow theory, where the sugar diffuses from a region of high sugar concentration to a region of low sugar concentration [127]. When the translocated sugar reaches a sucrose-storage region, usually the fruit, it is unloaded from the phloem tissue through a symplasmic or apoplastic pathway. The parenchymal cell in the fruit takes up the translocated sugars via a localized transporter on the plasma membrane [128].

In the cell cytoplasm, the translocated sugars are subjected to modification. First, the sucrose is broken down into fructose and glucose by invertase and sucrose synthase, sorbitol will be metabolized by sorbitol dehydrogenase, and then sucrose is re-synthesized again by sucrose phosphate synthase [129]. In addition, cell-wall turnover or arabinogalactan protein break down can cause the release of galactose in plants [130,131]. The final step is the compartmentalization, where the sugars are stored in the vacuoles. As sugars accumulate in the vacuoles, high osmotic pressure is produced, causing an influx of water into the vacuoles. Therefore, turgor pressure is created, which enlarges the parenchymal cells [128,129,132].

### 5.2. Glucose

Glucose is the main form of energy for most bacteria [133]. Glucose is a hexose containing six carbon atoms with a formula of C_6_H_12_O_6_. In an oxygen-rich state, the pyruvate generated in glycolysis undergoes oxidation, a citric acid cycle, and an electron transport chain. In oxygen-deprived environments, anaerobic bacteria perform glycolysis to generate adenosine triphosphate (ATP), an energy-storing molecule used for cellular respiration and reproduction. Simply put, the glycolytic pathway involves a series of steps to convert one glucose molecule into two pyruvate molecules. As a result, a net of two ATP molecules is generated from two adenosine diphosphate (ADP) molecules. Simultaneously, two reduced forms of nicotinamide adenine dinucleotide (NADH) are oxidized into two NAD^+^ molecules [134]. The glycolytic pathway can be summarized in the equation below:

C_6_H_12_O_6_ + 2ADP + 2P_i_ + 2NAD^+^ → 2CH_3_COCOO^−^ + 2ATP + 2NADH + 2H_2_O + 2H^+^

NAD^+^, however, is a limiting-factor molecule in glycolysis, as it is absent in the cell. Therefore, the lactic acid fermentation serves as an NAD^+^ regeneration process that recycles the electron acceptor NAD^+^ from the pyruvate through the lactic acid reduction process back into the glycolysis process for continuous ATP production. In lactic acid fermentation, two pyruvate molecules are reduced into two lactic acid molecules [135].

### 5.3. Fructose

Fructose is another subcategory of monosaccharides and is expressed as a six-carbon ketone sugar. While most bacteria utilize glucose, some lactic acid bacteria (LAB) have evolved to cope in fructose-rich niches. The bacteria that can metabolize fructose are termed fructophilic lactic acid bacteria (FLAB) which mainly consists of *Fructobacillus* spp. Kodama et al. [136] was the first to isolate FLAB strains from a flower, which sets the search for other FLAB strains onwards. In contrast with LAB, FLAB shows excellent growth on fructose-rich media and poor growth on glucose-rich media. In addition, all FLAB are classified as obligate heterofermenter, but, in contrast to LAB, they produce acetate instead of ethanol.

**Figure 3:**
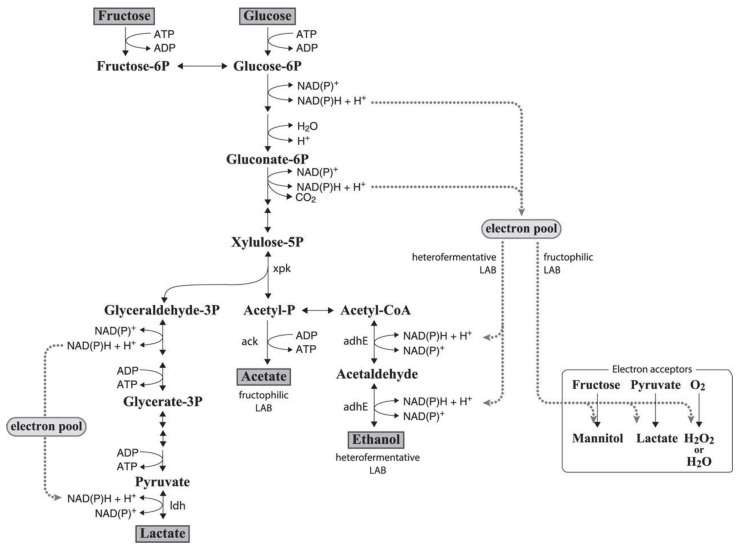
Utilization of glucose by fructophilic LAB. Adapted from Endo et al. [140].

Normally, an obligately heterofermentative LAB metabolizes glucose through the phosphoketolase pathway, but *Fructobacillus* spp. does not seem to express the *adh*E gene needed to produce the enzyme for conversion of acetyl phosphate to ethanol [137]. Moreover, the conversion of acetyl phosphate to ethanol contributes to the oxidation of NADH to NAD. Hence, the missing step in the NADH production upstream of the phosphoketolase pathway causes a shortage in NADH to metabolize sugar. Endo [138] revealed that *Fructobacillus tropaeoli* F214-1^T^ has the maximum growth curve on glucose under aerobic conditions, followed by glucose in the presence of pyruvate, on fructose media, and lastly the lowest growth curve on glucose. This finding suggests that pyruvate and oxygen are used as the external electron acceptor for FLAB to grow on glucose substrates. Furthermore, FLAB can also use phenolic acids as the external electron acceptors [139]. As seen in Figure 3, FLAB utilizes fructose, pyruvate, and oxygen as electron acceptors to produce mannitol, lactate, and water, respectively.

### 5.4. Galactose

Galactose is one of the three dietary six-carbon monosaccharides, along with glucose and fructose. LAB is known to metabolize lactose in milk and convert it into glucose and lactose. While glucose is readily used for glycolysis, galactose is less efficiently metabolized. LAB can take up and metabolize galactose in two ways via the Leloir pathway or PEP-transferase system (PTS). The first method involves the importation of galactose into the cell by permease GalP and subsequently channeling into the Leloir pathway reactions [141]. The reaction starts with the modification of β-d-galactose to α-d-galactose by galactose mutarotase enzyme (encoded by *galM* gene). Then, α-d-galactose is phosphorylated by galactokinase enzyme (encoded by *galK* gene) into galactose-1-phosphate. Next, one UMP group from UDP-glucose is transferred to galactose-1-phosphate by galactose-1-phosphate uridyltransferase (encoded by *galT* gene) to form UDP-galactose and glucose-1-phosphate. The Leloir pathway is completed by the interconversion between UDP-galactose and UDP-glucose by UDP-galactose-4-epimerase (encoded by the *ale* gene), where it can be used again to convert galactose-1-phosphate into glucose-1-phosphate [141]. The glucose-1-phosphate is converted into glucose-6-phosphate by phosphogucomutase and used in glycolysis.

**Figure 4:**
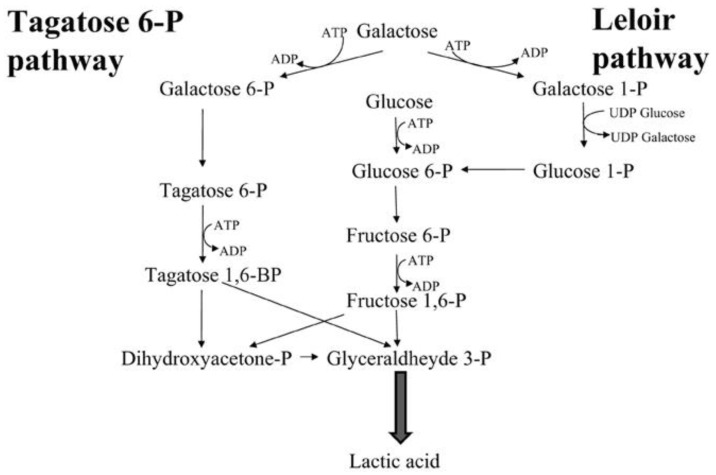
Interconnection between the Leloir and Tagatose-6-phosphate pathway in bacterial galactose metabolism. Adapted from Pessione, [143].

The second method of galactose metabolization in LAB is through the lactose-specific PTS^LacEF^ (happens in the presence of lactose) or galactose-specific PTS [141,142]. The intracellular 6-phospho-β-galactosidase (encoded by *lacG* gene) converts galactose into galactose-6-phosphate. The galactose-6-phosphate is fed into the tagatose-6-phosphate pathway. The tagatose-6-phosphate pathway starts from the isomerization of galactose-6-phosphate by the galactose-6-phosphate isomerase (encoded by *lacAB* gene) into tagatose-6-phosphate. Then, the tagatose-6-phosphate is phosphorylated into tagatose-1,6-bisphosphate by tagatose-6-phosphate kinase (encoded by *lacC* gene). The final step in the tagatose-6-phosphate pathway is the cleavage of tagatose-1,6-bisphosphate into a triose dihydroxyacetone-phosphate by tagatose-1,6-bisphosphate adolase (encoded by *lacD* gene) [141,142]. The dihydroxyacetone-phosphate is converted into the glyceraldehyde-3-phosphate of the glycolysis pathway by the triose-phosphate isomerase and becomes the precursor for lactic acid production. Figure 4 shows the two-pathway process in which galactose is used in the glycolytic pathway.

### 5.5. Sucrose

Sucrose is a disaccharide consisting of one unit of α-d-glucopyranose (glucose) molecule and one unit of β-d-fructofuranose (fructose) molecule with molecular formula, C_12_H_22_O_11_. The unique glycosidic bond is made between one end of reducing sugar and another end of non-reducing sugar inhibits sucrose from a spontaneous reaction with cellular and circulatory molecules. Furthermore, sucrose is a non-reducing sugar due to the absence of anomeric hydroxyl groups. Sucrose occurs naturally in fruits, vegetables, nuts, and commercial crops such as sugar cane and sugar beets [144].

The ability of LAB in sucrose metabolism depends on the expression of specific sucrose permease and/or sucrose-hydrolyzing enzymes. Bacteria can express two types of permeases, which are the phosphotransferase system sucrose-specific EII component (PTS-EII^scr^) and sucrose permease. The PTS-EII^scr^ modifies external sucrose into sucrose-6-phosphate and takes it up into the cytosol, while the non-PTS sucrose permease allows entry of the unmodified sucrose [145]. Additionally, bacteria can secrete sucrose-hydrolyzing enzymes such as β-fructofuranosidases and sucrose-6-phosphate hydrolases. The β-fructofuranosidases belongs to family with 32 members of the glycosyl hydrolase that catalyze the hydrolysis of high-molecular-weight fructose polymers. Meanwhile, sucrose-6-phosphate hydrolases also belongs to the same glycosyl hydrolase group but specifically to hydrolase low-molecular-weight fructoses such as sucrose and raffinose.

**Figure 5:**
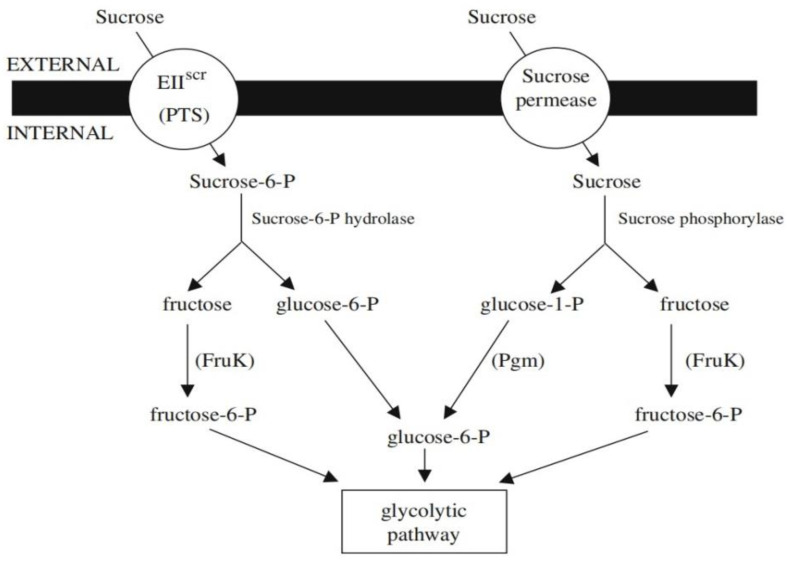
Sucrose metabolism producing intermediates for glycolytic pathway. Adapted from Shanton and Valerie, [145].

The sucrose-6-phosphate hydrolases causes the cleavage of sucrose-6-phosphate accumulated from the PTS system into glucose-6-phosphate and fructose. Another enzyme secreted is the sucrose phophorylases that belong to the family with 13 members of the glycosyl hydrolases [145]. The unmodified sucrose undergoes reversible phosphorolysis by the sucrose phophorylases in the presence of inorganic phosphate to form glucose-1-phosphate and fructose. However, the reversible phosphorolysis is regarded as an energy-saving cellular process, where it does not require ATP energy. The glucose-1-phosphate is then modified into glucose-6-phosphate by phosphoglucomutase (encoded by *Pgm* gene). Furthermore, an ATP-dependent fructokinase (encoded by *FruK* gene) will catalyze the phosphorylation of the sucrose-6-phosphate into fructose-6-phosphate. The glucose-6-phosphate and fructose-6-phosphate are the intermediate in the glycolytic pathway to produce lactic acid [145]. Figure 5 shows how the PTS system and sucrose permease facilitate the uptake of sucrose before being metabolized by sucrose-specific enzymes.

### 5.6. Fermentability of Vegetable and Fruit Juices by Lactic Acid Bacteria

Fermentation is a metabolic process which occurs in yeast and bacteria to convert sugar into alcohol, acids, and/or gases. In the fermentation industry, fermentation is referred to as the bulk growth of microorganisms in a bioreactor supplied with growth medium [146,147]. In the absence of oxygen, microbes perform fermentation to break down organic compounds into small amounts of ATP energy, enough to remain alive, while releasing by-products such as alcohol and organic acid [9]. These by-products have value in the beverage market. Depending on the substrate, alcoholic fermentation turns sugary liquid samples into alcoholic beverages such as wine, mead, beer, whiskey, rice wines, and rum. Similarly, lactic acid fermentation produces lactic acid, which lowers the pH of the substrate and enhances the flavor of the final product (tangy and sour taste profile depending on the acid concentration) [8]. Aside from enhancing the flavor, the by-product from microbial fermentation inhibits the growth of other opportunistic microorganisms, which increases the shelf-life of the product [148].

In general, there are two types of fermentation mainly applied in the fermentation industry, namely alcohol fermentation and lactic acid fermentation. Alcoholic fermentation is the oldest of all biotechnological applications. The key player in alcoholic fermentation is yeast (*Saccharomyces cerevisiae*) [149]. In an oxygen-deficit state (anaerobic), yeast performs alcoholic fermentation, which takes place in the cytosol [150,151]. In the cytosol, glycolysis occurs by causing a glucose molecule to be broken down into two pyruvate molecules (the ionized state of pyruvic acid). Then, the reduction of the two pyruvic acid molecules results in the formation of two ethanol molecules and two carbon dioxide molecules [9]. In contrast, lactic acid fermentation is performed by lactic acid bacteria (LAB) [152]. In the anaerobic state, LAB generates lactic acid from pyruvate molecules, the product of glycolysis. The purpose of lactic acid fermentation is to regenerate the electron carrier NAD^+^ by allowing the NADH to donate electrons to pyruvate, causing it to be reduced to lactate and oxidize NADH into NAD^+^. As a result, the glycolysis process can obtain a steady supply of NAD^+^ to generate ATP energy [153].

Lactic acid bacteria (LAB) are mostly Gram-positive, non-motile, and non-spore-producing with rod and cocci shapes. They are facultative bacteria that can grow both in aerobic and anaerobic condition (mostly anaerobic) [154]. LAB exhibits two types of fermentation pattern: homo fermentative LAB, which produces only lactic acid, and hetero fermentative LAB, which produces lactic acid, together with other metabolites such as carbon dioxide, short chain fatty acids, acetyldehyde, diacetyl, and ethyl alcohol [13,14]. Examples of LAB includes several genera from *Lactobacillus*, *Pediococcus*, *Lactococcus*, *Streptococcus*, *Enterococcus*, *Oenococcus*, *Leuconostoc*, and *Weissella*. Nonetheless, the genera *Lactobacillus* and *Bifidobacterium* are commonly used as probiotics [155], as both contain fewer pathogenic strains, produce antimicrobial compounds, and induce therapeutic effects in the consumer [16].

Most LAB are mesophiles which can grow at temperature range of 14 °C to 45 °C, where they grow best at optimal temperatures of 35 °C to 39 °C [154]. Some isolated LAB strains are reported to be psychrophiles and thermophiles. Kasimin et al. [156] isolated five strains of bacteria capable of producing antimicrobial substances from dairy products and raw milk, where two isolates, namely the *Lactobacillus* sp. strains CA1 and CA4, are able to grow at a temperature range of −20 °C to 37 °C (psychrophiles), while the other three antimicrobial-producing LAB, coded as CCB1, GB3, and CB3, were able to grow at the temperature range of 28 °C to 70 °C (thermophiles). Gangwar et al. [30] reported that the *Bacillus coagulans* MTCC 5856 strain, a commercialized spore-forming LAB is thermophilic in nature (growth at 52 °C).

In the fermentation of non-dairy matrices, probiotic strains are reported to follow the same sigmoid growth curve pattern, measured as a colony-forming unit (CFU) per gram (g) or milliliter (mL) or cell density of the sample as shown in Table 4. Yuliana et al. [18] reported that the growth curve of *L. acidophilus* first shows the lag phase where the viable cell count remained around 4 log CFU/mL from 0 to 4 h of fermentation in coconut milk at 37 °C. Between 4 and 20 h, the growth curve became exponential, where the viable cell count increased from 4 log CFU/mL to a maximum of 9.89 log CFU/mL. The *L. acidophilus* retained its maximum cell viability until the 28 h fermentation period was over.

## 6. Quality Indicators of Fermented Product

### 6.1. Total Soluble Solids and Sugar Consumption

The total soluble solid (TSS) is a measurement of the sugar content inside the sample. The TSS value of a sample solution is measured using a refractometer and determined by the index of refraction. The unit of TSS is degrees Brix (°Brix) [158]. Meanwhile, the total sugars and reducing sugar are measured via the phenol-sulfuric method [30]. In all of the fermentation process, LAB utilizes sugars in the media for growth, respiration, and reproduction. Sugar is used in the glycolytic pathway to generate pyruvate and ATP energy. The pyruvate will be metabolized in the lactic acid fermentation to regenerate NAD^+^ for the continuous production of ATP in the glycolysis process. The increased number of bacterial cells will replace and add value to the TSS. The refractive index increases due to the slower travel time of light as the cell density increases [161].

The continuous depletion of fermentable sugars will slow down the exponential growth of the bacteria. At plateau, almost all sugars are depleted, causing a rapid decrease in viability, and the cell enters the death phase due to accumulation of waste. Gangwar et al. [30] noticed a decrease in the total sugars and reducing sugar from 3.96% and 2.37% to 3.15% and 2.27%, respectively, after two days of fermentation with *Bacillus coagulans* and an increase in total soluble solids from 5.0 to 6.0 °Brix. The increase in TSS value is caused by the increased viable cell counts of the probiotics, leading to an increase in the refractive index of the sample from 1.340 to 1.342. A reduction in sugar causes the end-product of a fermented probiotic juice to be less sweet than the unfermented fruit juice [30,69].

### 6.2. Titratable Acidity and pH

Titratable acidity is the measurement of total acids inside a food sample [162]. Titratable acidity is expressed as a percentage unit of the major organic acid present in the food and measured via the titration method with sodium hydroxide (NaOH) and phenolphthalein as the indicator. In the case of fruit and vegetable matrices, the titratable acidity is written as % citric acid, as citric acid is mostly found in this sample [163]. In contrast, when these matrices are incubated with lactic acid bacteria, the acidity of the final fermentation product is measured as % lactic acid, because lactic acids are produced as the main product of lactic acid fermentation [164]. The titratable acidity and pH value of the sample are interrelated. The titratable acidity measures the total amount of acids, while the pH measures the strength of the acids.

Acid productions (primarily lactic acid) occur after the consumption of the sugar in the fruit and vegetable juice by the LAB. As the sugars are consumed, more lactic acids are produced, causing an increase in the H^+^ concentration and a decrease in the pH value of the sample [18,19,30]. The decrease in pH favors the growth of lactic acid bacteria [154], which are also known to be acidophilic [165]. The LAB from the genera *Streptococcus* and *Leuconostoc* can tolerate an acidic pH range from 4.0 to 4.5, while some species of *Lactobacilli* and *Pediococci* grow around pH 3.5 [166]. Yuliana et al. [18] reported that, during the 28 h fermentation period of coconut water by *L. acidophilus*, there was an inversely proportional relationship between the titratable acidity and the pH. At the end of the fermentation period, *L. acidophilus* retained a high viable cell count at 9.89 log CFU/mL at final pH of 3.87. Similarly, Yoon et al. [19] revealed that there was an overall decrease in the pH of cabbage juice from 5.4 to 3.5 when the acidity (% lactic acid produce) increased from 0.12% to 0.86% lactic acid by *L. plantarum*, *L. casei*, and *L. delbrueckii*.

Regardless of how remarkably tolerant the LAB are to the acidic environment, there is a certain threshold of pH values which, when exceeded will cause damage to the cells. As the fermentation media accumulate more acids, the negative feedback mechanism slows down the growth of LAB to prevent the over-acidification of the cell cultures [167,168,169]. As such, the viable cell count reaches its maximum at its lowest tolerable pH, depending on the species and strain of the LAB [170]. Furthermore, most enzymes of LAB functions best in neutral environments. The phosphofructokinases of *Lactobacillus bulgaricus* require an optimum pH of 8.2 to function properly [171]. A neutral pH ranging from 6.9–7.5 is required by the pyruvate kinase of *L. lactis* ssp. lactis to catalyze the conversion of phosphoenolpyruvate and ADP to pyruvate and ATP in glycolysis [25]. The aminopeptidase of *L. casei* requires a near-neutral pH of 6.5 to cleave amino acids from the *N*-terminus or proteins [172].

Thus, LAB will perform pH homeostasis to cope with the increasing acidity of the fermentation medium [173]. To maintain a more alkaline cytoplasmic environment compared to the surrounding, LAB cells will perform a carrier-mediated process to rapidly remove protonated lactic acid from the extracellular medium [174,175]. Consequently, the cellular membrane of the LAB becomes impermeable to extracellular protons, including lactate molecules produced during fermentation. A pH gradient (Δ pH) is formed, where there are differences between the external pH (pH_out_) and internal pH (pH_in_) values, generating a proton motive force [176]. Balanced pH_out_ and pH_in_ (pH_out_ = pH_in_) values allow the maintenance of Δ pH and pH homeostasis to be performed for optimal growth. A lowering of the external pH (pH of the medium) compared to the internal pH (pH of the cytoplasm) drives the proton motive force of H^+^ influx into the cell [173]. Therefore, to raise the pH_in_, LAB such as *Streptoccci*, *Lactocci*, and *Lactobaclli* have a proton symport system called H^+^-ATPase to extrude protons out of the cell by ATP hydrolysis. This membrane-bound proton-translocating enzyme exports protons from the alkaline cytoplasm to the acidic fermentation medium against the concentration gradient of protons, thereby requiring energy in the form of ATP [177].

### 6.3. Stability at Low Storage Temperature

Refrigeration is a way to extend the preservation of food. According to the U.S. Food and Drug Administration (FDA), the basic guidelines for storage include a working refrigerator temperature at or below 40°F (4 °C), while the freezer compartment should be 0 °F (−18 °C). Therefore, all household and laboratory refrigerator temperatures should operate at 4 °C as a means to prevent or slow down the growth of foodborne pathogens such as *Salmonella*, *L. monocytogenes*, and *C. botulism*. Low temperatures slow down metabolism and extend the log or stationary phase of the mesophilic bacterial growth curve [154].

Most LAB are mesophiles, while some exhibit cold-tolerant characteristics. Psychrophiles are microorganisms capable of growing at very low temperatures ranging from −12 °C to 20 °C, where the optimum temperature of 15 °C shows the best growth [154]. However, most psychrophiles tend to die when exposed to mild mesophilic temperatures. Major psychrophilic organisms originate from the Antarctic and Artic regions; these includes *Bacillus psychrophilus*, *Chlamydomonas nivalis* and *Polaromonas vacuolate* [178]. Hence, microorganisms need to adapt at near-zero temperatures to survive and sustain their cell cycle [179]. The adaptation at low temperatures features the synthesis of cold-active enzymes [180] and adequate translation and proper protein folding [181].

At low storage temperatures, researchers find it hard to maintain an ideal number of viable cells of their probiotic strains. Therefore, the selection of potential probiotic strains with the psychrophilic trait would be beneficial for the storage of probiotic beverages. Kasimin et al. [156] successfully isolated two psychrophilic antimicrobial-producing LAB isolated from fresh raw milk obtained from the Department of Veterinary Services. The isolated *Lactobacillus* sp. CA1 and CA4 can grow at a temperature range of −20 °C to 37 °C. The adaptation of the isolated LAB strains at low temperatures may be due to the storage of the milk inside the refrigeration temperature at the veterinary department. Milk samples are stored to preserve taste, texture, and aroma while halting the growth of pathogens. The correlation of storage temperature and adaption of bacteria to the cold environment is supported by the findings of Kato et al. [182]. They isolated *Lactobacillus algidus* sp. nov. from vacuum-packaged refrigerated beef, which exhibited a psychrophilic nature due to its ability to grow on MRS agar at 0–25 °C, but no viable cells were detected at 30 °C.

Additionally, several studies on probioticated non-dairy beverages showed that some probiotic strains are capable of retaining an appreciable number of viable cells at low storage temperatures. To exert therapeutic effects, the probiotic drink should contain a minimum of 10^6^–10^7^ CFU/mL [2,3]. Yoon et al. [19] discovered that the ability to remain viable at a refrigeration temperature of 4 °C is strain dependent. After 3 days of fermentation on cabbage juice, *L. casei*, *L. plantarum*, and *L. delbrueckii* accumulated viable cell counts of 1.1 × 10^9^ CFU/mL, 17.5 × 10^8^ CFU/mL, and 11.0 × 10^8^ CFU/mL, respectively. In cold storage at 4 °C, however, it was observed that *L. casei* dramatically decreased in viability after one week of storage (1.1 × 10^6^ CFU/mL) before cell death in the following weeks. On the other hand, *L. delbrueckii* retained the ideal viable cell count of the probiotic dose for two weeks (10^7^–10^8^ CFU/mL) but decreased gradually at week 3 (34.3 × 10^5^ CFU/mL) and week 4 (4.5 × 10^5^ CFU/mL). The study discovered that the viable cell count of *L. plantarum* remained at an ideal daily probiotic dose around 10^7^–10^8^ CFU/mL.

Praia et al. [159] revealed that the biochemical constitution of the non-dairy substrate can affect the stability of probiotics at storage temperature. They discovered that *Lactobacillus casei shirota* retained the ideal range of probiotic dose (10^8^–10^9^ CFU/mL) under refrigeration temperature 5 °C to 8 °C for 24 to 96 h in fresh coconut water compared to industrialized coconut water, where the probiotic viability started to decline after 72 h and reached an almost-zero viable cell count at 96 h storage. They believed that the industrial processing and addition of antioxidants altered the nutritional profile of the substrate, influencing the pH stability and acidity.

## 7. Conclusions

The development of plant-based probiotic drinks is important in promoting healthier alternatives to dairy-based drinks. A thorough grasp of the range of plant fermentable sugars, as well as the critical quality indicators of fermented products, aids in the production of high-quality functional beverages. The difficulties in maintaining the high viability of probiotics in fruit-and-vegetable-based drinks impose challenges to meet the increasing demand by consumers. The commercialization of non-dairy probiotic drinks creates a variety of taste options and promotes healthier lifestyles for consumers [172]

## Figures and Tables

**Table 2:** List of non-dairy probiotic products sold commercially.

Product Name	Manufacturer	Probiotic Strain (s)	Non-Dairy Substrate
Biomel	Biomel, UK	*B. bifidum*, *B. coagulans*, and *L. plantarum*	Coconut milk and grape extract
Califia Farms	Califia Farms, California	*Bifidobacterium* BB-2, *S. thermophilus*, and *L. bulgaricus*	Almond milk, coconut cream, and oat fiber
GT’s Organic Kombucha	GT, Los Angeles	Lactobacillus bacterium (species not specified) and *Bacillus coagulans* GBI-30 6086	Black and green tea (to make Kombucha) and kiwi juice
KeVita Apple Cider Vinegar Tonics	KeVita, California	Water kefir (starter culture) and *Bacillus coagulans* GBI-30 6086	Apple juice (to make apple cider), apple juice, and lemon extract.
Plantiful	Lifeway Foods Inc., Illinois, U.S.	*L. casei*, *L. plantarum*, *B. bifidum*, *B. animalis sub*sp. *lactis*, *B. longum sub*sp. *longum*, *L. acidophilus*, *L. paracasei*, *L. rhamnosus*, *L. lactis sub*sp. *lactis*, and *S. thermophilus*.	Non-GMO pea protein
GoodBelly JuiceDrink	NextFoods, Boulder, Colorado	*Lactobacillus plantarum* LP299V	Mango: pear juice, mango puree, banana puree, oat flour, barley malt Cranberry watermelon: grape juice, pear juice, cranberry juice, strawberry juice, oat flour, watermelon juice, barley malt, vegetable juice
Harmless Harvest Dairy-Free Yogurt	Harmless Harvest, Thailand	*L. acidophilus*, *B. lactis, S. thermophilus*, *L. casei*, *L. bulgaricus*, *B. bifidum*, *L. rhamnosus*, and *Bifidobacterium lactis* HN019	Young Thai coconut milk and water
Tropicana Essentials Probiotics^®^ Pineapple Mango	PepsiCo, U.S.	*Bifidobacterium lactis*	Mango puree, pineapple, banana puree, and vegetable juice
VitaCup Immunity Coffee Pods	VitaCup, San Diego	*Bacillus coagulans*	Coffee, Inulin
Gut Shot^®^	Farmhouse Culture	Naturally occurring bacteria in the cabbage (Not specified)	Sauerkraut brine (fermented cabbage) and apple
Dee-V Drinks	Dates Valley, Malaysia	Not specified	Khal dates cider with four optional flavors (honey, berry, ginger, lemon)
Gut Kulture	Steve’s PaleoGoods, New Jersey	Naturally occurring probiotics culture (Not specified)	Beet, carrot, sarsaparilla, turmeric, ginger, burdock root, kudzu root, astragalus root, shatavari root, dandelion root, white ginseng, ashwaganda, rhodiola root

**Table 3:** Advantages and disadvantages of cereal and fruit-based probiotic beverages.

Probiotic Beverages	Advantages		Disadvantages		
	Healthier Alternative	Wider Taste Selection	Stability/Shelf Life	Nutrition	Production Process
Cereal-based	Contains carbohydrates, proteins, dietary fibre, minerals, and vitamins with lesser fat content and cholesterol	Can be made from different source of cereal such as rice, millet, oat, and barley.	Stability in refrigeration period depends on strain of probiotic and type of cereal.	Exposed to starch gelatinization and increased viscosity	Complex: Involving various step in preparation of cereal milk (soaking, draining, wet milling, heat treatment, colling) followed by fermentation with probiotic strain and products formulation.
Fruit-based	Different types of fruit contain handful amount of carbohydrates, proteins, dietary fibres, minerals, and vitamins. Most fruit contain bioactive compound with antimicrobial, antioxidant, and anticancer properties	Can be made from different type of fruit ranging from sweet to citrusy fruit. Taste and aroma of each fruit differs depending on type and maturation stage.	Stability in refrigeration period depends on strain of probiotic and type of fruit.	Exposed to oxidation of the antioxidant (ascorbic acid)	Depends on type and parts of fruit: Fruit such as coconut with hard shell need to be de-husked and cracked open to obtain coconut water. The flesh needs to be grated and pressed to obtain coconut milk. Easier fruit such as grapes are only pressed to release the juice and introduced into heat treatment before inoculation and fermentation by probiotic strain.

**Table 4:** Change in viable cell count of probiotics grown on fruits and vegetable matrices.

Non-Dairy Substrate	Probiotic	Change in Cell Density/Viable Cell Count	Reference
Coconut water	*Bacillus coagulans* MTCC 5856 spore	After 2 days of fermentation the cell density of *Bacillus coagulans* at 540 nm increased from 0.121 to 0.683, corresponding to viable cell count of 10^9^ CFU/mL (cell density higher than 0.600).	[30]
	*Lactobacillus acidophilus* L10 and *Lactobacillus casei* L26	Both strains showed increase in viable cell count during the 2 days’ fermentation where *L. acidophilus* showed higher growth at 3.58 × 10^8^ CFU/mL compared to *L. casei* at 1.41 × 10^8^ CFU/mL on day 2.	[157]
Coconut water + inulin	*Lactobacillus plantarum* BG 112	The viable cell count of 9 log CFU/mL of the starting inoculum dropped to a range of 6.00 to 8.70 log CFU/mL depending on the temperature after 16 h of fermentation. Fermentation at temperature 32 °C supplied with 0.5% (*w*/*v*) inulin retained the highest viable cells at 8.85 log CFU/mL.	[158]
Industrialized and fresh coconut water	*Lactobacillus casei shirota*	After 48 h of fermentation at 36 °C, *Lactobacillus casei shirota* experienced an increase in viable cell count from 4.15 × 10^7^ CFU/mL to 7.56 × 10^8^ CFU/mL in industrialized coconut water but in fresh coconut water, the cell count increased from 5.4 × 10^7^ CFU/mL (0 h) to 2.5 × 10^9^ CFU/mL (6 h) but no observable cell colony from 18 h to 48 h incubation period.	[159]
Coconut milk	*Lactobacillus acidophilus*	The viability of *L. acidophilus* at initial viable cell count log 4.32 CFU/mL increased after 4 h of fermentation in coconut milk at 37 °C and reached maximum viable cell count of log 9.89 CFU/mL at 20 h and remain constant until 24 h.	[18]
Breadfruit supernatant	*L. acidophilus, L. casei*, and *L. plantarum* DPC 206	*L. acidophilus, L. casei*, and *L. plantarum* DPC 206 showed increased in viable cell count from 5.275 to 8.029 log CFU/mL, 6.055 to 7.952 log CFU/mL, and 5.555 to 7.764 log CFU/mL, respectively, after 72 h of fermentation in breadfruit supernatant at 37 °C.	[69]
Mango juice and sapota juice	*Lactobacillus plantarum* NCDC LP 20	Increased viable cell count from 10^5^ CFU/mL to 8.1 × 10^8^ CFU/mL in mango juice and to 8.0 × 10^8^ CFU/mL in sapota juice after 72 h incubation at 30 °C.	[68]
Cabbage juice	*L. casei* A4, *L. debrueckii* D7 and *L. plantarum* C3	*L. casei* A4, *L. debrueckii* D7, and *L. plantarum* C3 showed increase in viable cell count from 3.0 × 10^6^ to 11 × 10^8^ CFU/mL, 4.3 × 10^5^ to 11 × 10^8^ CFU/mL, and 8.0 × 10^5^ to 7.05 × 10^8^ CFU/mL, respectively, after 48 h incubation at 30 °C.	[19]
Watermelon and tomato juice	*L. fermentum* and *L. casei*	Each probiotic shows excellent growth at 37 °C in watermelon and tomato juice combination where *L. fermentum* and *L. casei* showed increase in viable cell count from 4.6 × 10^7^ to 2.3 × 10^8^ CFU/mL and 2.7 × 10^7^ to 9.4 × 10^8^ CFU/mL, respectively, after 72 h of fermentation.	[160]
Pomegranate juice + grape juice + tomato juice + pomegranate peel extract	*L. plantarum* and *L. delbrueckii*	When the pomegranate juice is combined with 10% (*v*/*v*) grape juice, 5% (*v*/*v*) tomato juice, 0.1% (*v*/*v*) pomegranate peel extract and added with 2.0 g/L glucose, both strains achieved the highest survival rate with cell count 4.74 × 10^6^ CFU/mL and 4 × 10^6^ CFU/mL of *L. plantarum* and *L. delbrueckii*, respectively.	[67]

## Data Availability

Not applicable.
